# Posterior unilateral approach with 270° spinal canal decompression and three-column reconstruction using double titanium mesh cage for thoracic and lumbar burst fractures

**DOI:** 10.3389/fsurg.2022.1089697

**Published:** 2023-01-11

**Authors:** Lei Shi, Qi-jun Ge, Yun Cheng, Lu Lin, Qing-Shuai Yu, Si Cheng, Xiao-Lin Chen, Hong-Quan Shen, Fu Chen, Zheng-jian Yan, Yang Wang, Lei Chu, Zhen-Yong Ke

**Affiliations:** ^1^ Department of Spine Surgery, The Second Affiliated Hospital of Chongqing Medical University, Chongqing, China; ^2^ Geriatric Clinical Research Center of Chongqing, Chongqing, China; ^3^ Jiangjin Central Hospital, Chongqing, China

**Keywords:** spinal canal decompression, three-column reconstruction, double titanium mesh cage, posterior approach, thoracic and lumbar burst fracture

## Abstract

**Objective:**

To evaluate the clinical effects of the posterior unilateral approach with 270° spinal canal decompression and three-column reconstruction using double titanium mesh cage (TMC) for thoracic and lumbar burst fractures.

**Materials and methods:**

From May 2013 to May 2018, 27 patients with single-level thoracic and lumbar burst fractures were enrolled. Every patient was followed for at least 18 months. Demographic data, neurologic status, back pain, canal compromise, anterior body compression, operative time, estimated blood loss and surgical-related complications were evaluated. Radiographs were reviewed to assess deformity correction, anterior body height correction, bony fusion and TMC subsidence.

**Results:**

The average preoperative percentages of canal compromise and anterior body height compression were 58.4% and 50.5%, respectively. All surgeries were successfully completed in one phase, the operative time was 151.5 ± 25.5 min (range: 115–220 min), the estimated blood loss was 590.7 ± 169.9 ml (range: 400–1,000 ml). Neurological function recovery was significantly improved except for 3 grade A patients. The preoperative visual analog scale (VAS) scores for back pain were significantly decreased compared with the values at the last follow-up (*P* = 0.000). The correct deformity angle was 12.4 ± 4.7° (range: 3.9–23.3°), and the anterior body height recovery was 96.7%. The TMC subsidence at the last follow-up was 1.3 ± 0.7 mm (range: 0.3–3.1 mm). Bony fusion was achieved in all patients.

**Conclusion:**

The posterior unilateral approach with 270° spinal canal decompression and three-column reconstruction using double TMC is a clinically feasible, safe and alternative treatment for thoracic and lumbar burst fractures.

## Introduction

Vertebral burst fracture is described as compressed fracture of the anterior and middle portions of the spinal column combined with mild to severe epidural compression by bone fragments that shifted into the spinal canal (1). They are commonly caused by severe trauma, including a fall from a height and traffic accidents (2). Vertebral burst fractures account for 10%–20% of all spine fractures. They frequently occur at the thoracic and lumbar junction and may cause neurologic complications and angular deformities (3).

The goal of surgical intervention for thoracic and lumbar burst fractures is to decompress the neural elements, stabilize the spinal column, restore vertebral body height, and correct angular deformities. Decompression and reconstruction can be achieved *via* anterior and posterior approaches for thoracic and lumbar burst fractures. However, the clinical results of fixation to correct kyphotic deformities *via* the anterior approach are obviously inferior to those of the posterior approach (3–5). Additionally, higher morbidity has been reported during the exploration of thoracic and lumbar vertebral bodies or discs *via* anterior transthoracic or retroperitoneal approach (6). The single-stage transpedicular or costotransversectomy approach for corpectomy and reconstruction of thoracic and lumbar burst fractures has been well described (3–5, 7). The approach allows not only circumferential decompression but also simultaneous posterior instrumentation. However, the posterior spinal structure is often completely removed for adequate decompression, thus losing the feasibility of posterior column fusion. Additionally, a large ventral supporting cage is usually required to reconstruct the anterior column, and its placement is technically demanding, carrying risk of injury to the surrounding neural elements. Especially in the lumbar spine, nerve root damage can cause lower limb dysfunction. In contrast, a small cage has insufficient bone graft contact area, which will increase the risk of non-fusion. Moreover, the pressure increase in the upper and lower endplates may lead to subsidence or endplate fracture. Therefore, a more convenient, safe, and alternative method for decompression and reconstruction of thoracic and lumbar burst fractures is needed.

## Materials and methods

We received permission from the ethics committee of our hospital and written informed consent from all the patients.

### Patients' characteristics and details regarding the evaluation methods

Inclusion criteria: (1) Single-level burst fractures with nerve injury located in the thoracic and lumbar and lumbar vertebrae, (2) Load Sharing Score (LSS) ≥6 points (8), (3) The thoracic and lumbar injury severity score (TLICS) ≥4 points. Exclusion criteria: (1) The fracture is located in the upper thoracic vertebra, (2) Pathological fracture due to tumor and infection. Data from 27 patients meeting the above criteria were retrospectively collected. All patients underwent a posterior unilateral approach for 270° spinal canal decompression and three-column reconstruction using double TMC between May 2013 and May 2018. The demographic data, cause, and level of the injury (Table 1), Visual Analog Scale (VAS) for back pain, neurologic status, operative time, estimated blood loss, surgical-related complications, and follow-up time were collected. Radiographs were reviewed to assess canal compromise, anterior body height compression, kyphotic/lordotic angle, anterior body height recovery, correct deformity angle, loss correct angle, bony fusion and TMC subsidence.

**Table 1:** Patient demographics.

Patient	Age	Gender	Injury level	Injury cause	Follow-up (months)
1	56	M	L1	Fall	26
2	25	M	L4	Fall	18
3	49	M	T12	Fall	21
4	60	M	T12	Fall	40
5	46	F	T12	Traffic accident	26
6	54	M	T12	Slip	18
7	61	M	L1	Traffic accident	26
8	46	M	L4	Traffic accident	19
9	51	F	T12	Fall	40
10	48	M	L3	Fall	24
11	55	M	L2	Fall	18
12	42	M	T12	Fall	24
13	35	M	T12	Fall	20
14	47	M	L1	Fall	20
15	46	M	L1	Impact	48
16	71	F	T11	Slip	36
17	45	F	L1	Fall	20
18	52	M	T12	Impact	46
19	33	M	L2	Fall	18
20	58	M	T12	Fall	20
21	61	M	L1	Traffic accident	26
22	65	F	T12	Fall	28
23	43	M	T11	Fall	25
24	47	M	T10	Fall	21
25	46	M	T12	Fall	23
26	36	M	L1	Traffic accident	20
27	59	M	L3	Fall	26

### The clinical and radiographic indexes were described as follows

**Figure 1:**
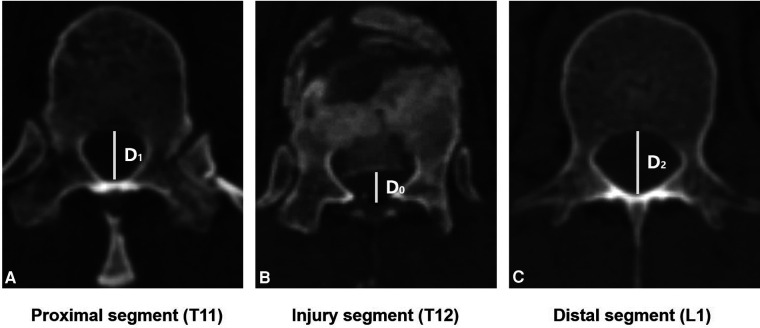
The midsagittal diameter of spinal canal measurement. D0 was the midsagittal diameter of the injured level's spinal canal (T12). D1 and D2 were the midsagittal diameters of the spinal canal at the levels above (T11) and below (L1) the injured level.

**Figure 2:**
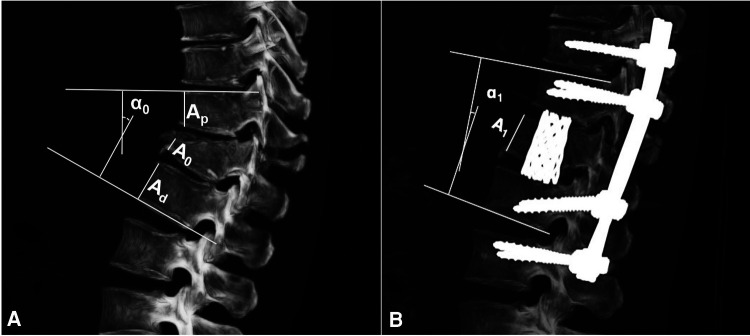
The measurement of angular deformity and anterior body height compression. *A*_0_ was the anterior body height of injured level; *A_p_* and *A_d_* was the anterior body height of the proximal and distal level; *A*_1_ was the anterior body height of injured level after surgery; *α*_0_ was pre-operative angle and *α*_1_ was post-operative angle of deformity.

**Figure 3:**
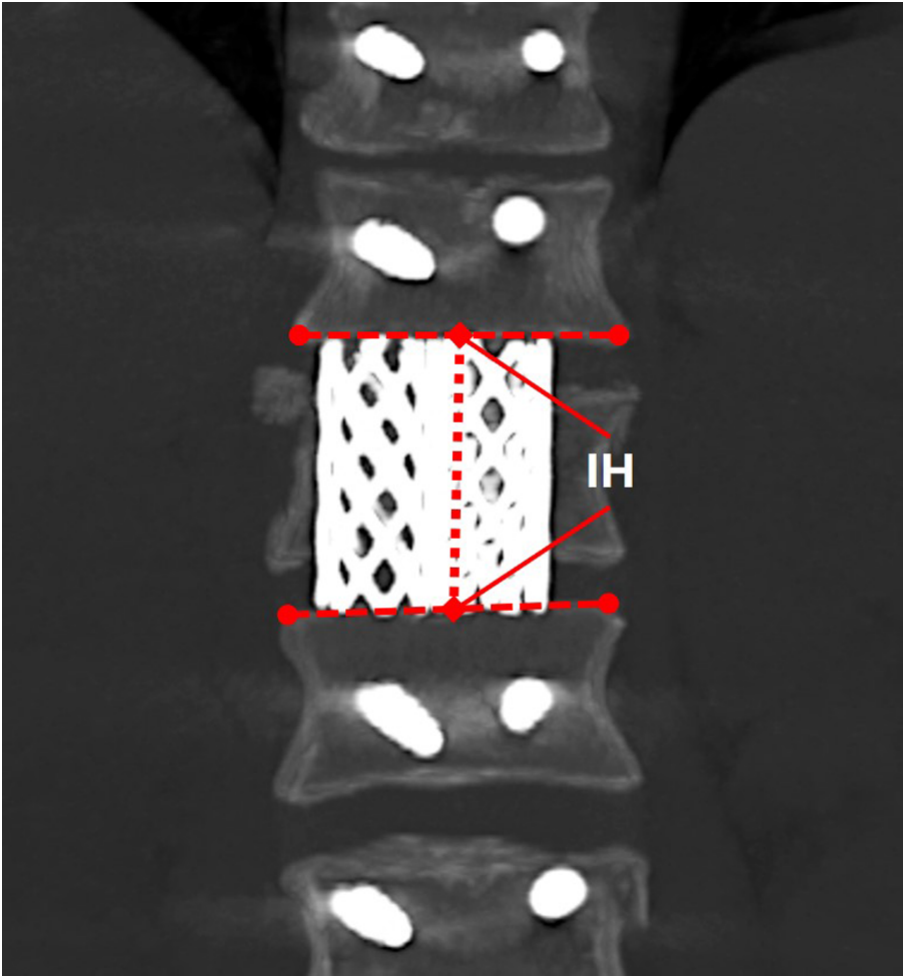
The intervertebral height (IH).

1. Neurologic status was assessed by the American Spinal Injury Association (ASIA) impairment score.
2. Canal compromise was calculated with the following formula: $a=(1-((2*D_{0})/D_{1}+D_{2}))*100\%$, where a was the percentage of canal compromise (5); *D*_0_ was the midsagittal diameter of the spinal canal of the injured level; and *D*_1_ and *D*_2_ were the midsagittal diameters of the spinal canal at the levels above and below the injured level (Figure 1).
3. Anterior body height compression was calculated with the following formula: $b_{0}=(1-((2*A_{0})/(A_{p}+A_{d})))*100\%$, where *b*_0_ was the percentage of anterior body height compression (9); *A*_0_ was the anterior body height of the injured level; and *A_p_* and *A_d_* were the anterior body heights of the proximal and distal levels (Figure 2).
4. Anterior body height recovery was calculated with the following formula: $b_{1}=((2*A_{1})/(A_{p}+A_{d}))\;*100\%$, where *b*_1_ was the percentage of anterior body height recovery; and *A*_1_ was the anterior body height of the injured level after surgery (Figure 2).
5. The kyphotic/lordotic angle (α) was the angle of kyphotic or lordotic deformity of the injured level, measured as the angle between the superior endplate of the vertebral body above the affected level and the inferior endplate of the vertebral body below the affected level (5) (Figure 2). *α*_0_ was the preoperative angle, and *α*_1_ was the postoperative angle of deformity. The kyphotic angle was defined as the positive value, and the lordotic angle was defined as the negative value.
6. Correct deformity angle (°): kyphotic correction = *α*_0_**−***α*_1_; lordotic correction = ***α***_**1**_**−*****α***_**0.**_
7. The correct loss angle was the reductive angle between the last follow-up and immediate post-operation.
8. Definition of bone fusion: the *x*-ray or CT film lacked lucency at the cage-vertebra junction, or the presence of mature bony trabeculae around the cage bridging the adjacent vertebral bodies was accepted as a sign of fusion (10, 11).
9. TMC subsidence was the total subsidence of intervertebral height (IH) from immediate post-operation to the last follow-up. IH was the distance between the midpoints of the inferior endplate of the proximal level and the superior endplate of the distal level (Figure 3). Subsidence over 2 mm or a correct loss angle over 4° was considered the presence of TMC subsidence in this study (12).

### Preoperative preparation

Preoperative radiologic tests included conventional radiography, thin-slice computed tomography, and magnetic resonance imaging. Neurologic status was also evaluated. The values of canal compromise (%), anterior body compression (%), and kyphotic/lordotic angle in CT reconstructed images were calculated. All radiographic parameters were measured with the DICOM Viewer System (RadiAnt, Medixant, Poland).

### Surgical procedures

All surgeries were performed by the same surgeon. The patient was placed in the prone position under general anesthesia. A midline incision was made to expose the laminae and facet joints two levels above and below the injured level. Muscle dissection was performed using an electrome until the bilateral facet joints were exposed. Then, the injured vertebra was confirmed by intraoperative C-arm fluoroscopy. Pedicle screws were introduced two levels above and below the injured level if there was no affiliated injury (3). The side of decompression was determined by the severity of canal compromise at the injured level. The screws were distracted axially, first on the side of decompression and then on the opposite side. Generally, semi-laminectomy was performed by using a high-speed drill or osteotome. Under visualization of the dural sac, the facet joint and pedicle of the same side were removed conventionally to expose the nerve root. Transverse processes in the lumbar and small section rib in the thoracic vertebra should be removed for better exposure to neural elements. Epidural veins and radicular veins were cauterized with bipolar forceps to avoid massive bleeding. In some cases, the spinous process should be removed as required for repair of dural sac rupture due to severe burst fracture first. Thereafter, adjacent intervertebral discs were completely resected. Then, a subtotal corpectomy of the fractured vertebral body was performed using osteotome and bone curette, and the lateral and anterior parts of the vertebral body wall were preserved in place. During the subtotal corpectomy, the ipsilateral and ventral cancellous bones were removed firstly. Thereafter, an off-centered curette was used to carefully scrape the bone tissue from ventral to dorsal. After the fragmented vertebral body of the posterior part had collapsed, then the bone fragments were carefully dissected by the off-centered curette. After decompression was completed, a corridor was created at the lateral edge of the vertebra to insert the TMC. The medial border of corridor was the dural sac, the lateral wall was the pleura or peritoneum, the inferior border was the corresponding nerve root, and the superior border was the lower endplate of the upper level.

**Figure 4:**
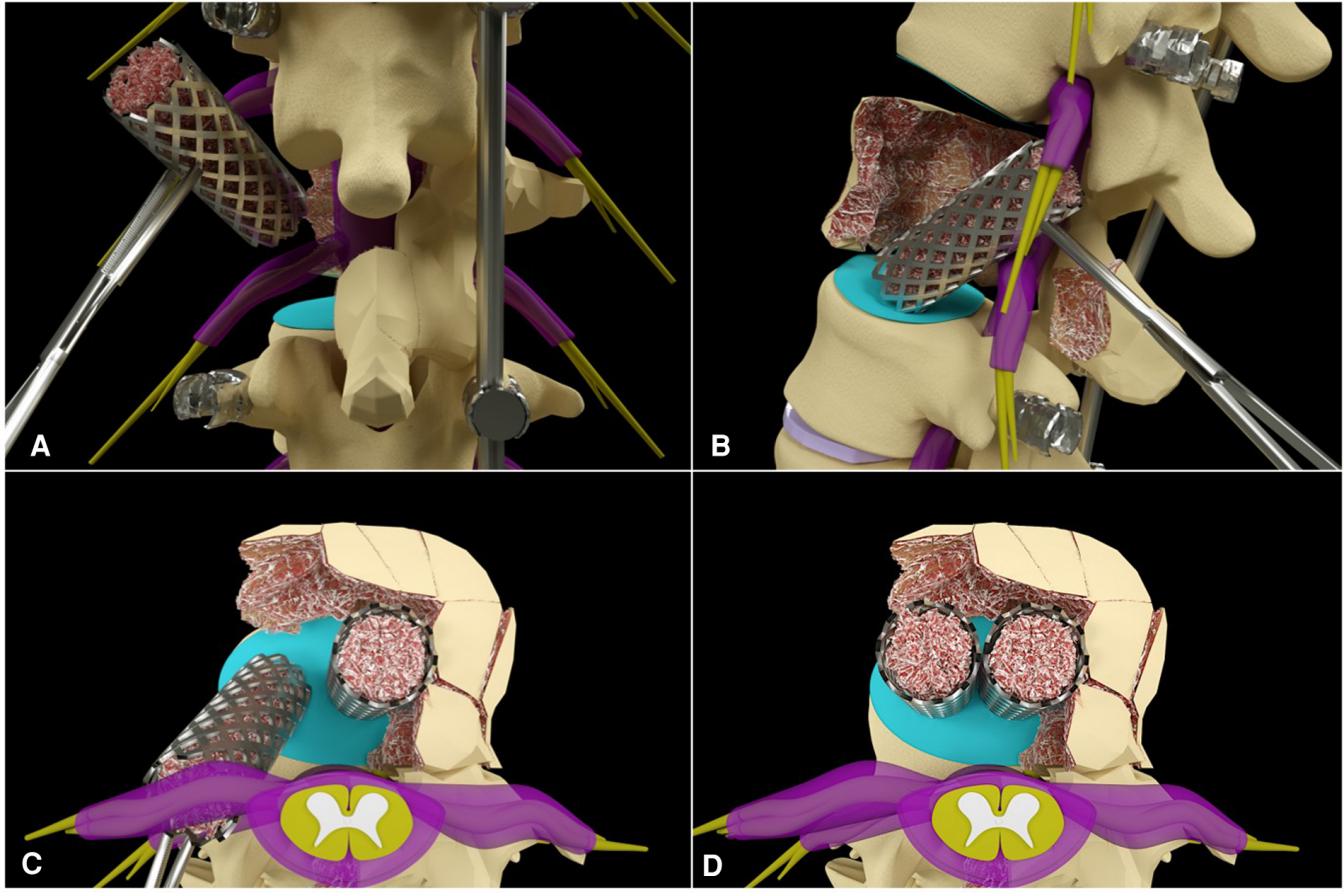
The schematic pictures of double TMC insertion.

Commonly, double TMC (WEGO Co., Weihai, Shandong, China) 16 mm in diameter were stuffed with chipped autograft bone. Then, the cage with appropriate length was inserted obliquely into the corpectomy defect through the corridor without any traction of the nerve roots or the dural sac (Figure 4A). Once inside the corpectomy defect, the cage was rotated to its proper position using the bone tamp to ensure sufficient contact and a tight fit against the inferior endplate of the upper vertebra (Figure 4B). The first cage should be placed as close to the opposite side as possible to reserve enough space to insert the second cage. Additionally, two cages could be placed on the same coronal plane for better balance (Figures 4C,D). After screw-rod compression, the cortex of the contralateral lamina and upper and lower facet joints were drilled, and the chipped autograft bone was covered for posterior column fusion. Eventually, the incised wound was sutured after sufficient and careful hemostasis.

### Postoperative management

**Figure 5:**
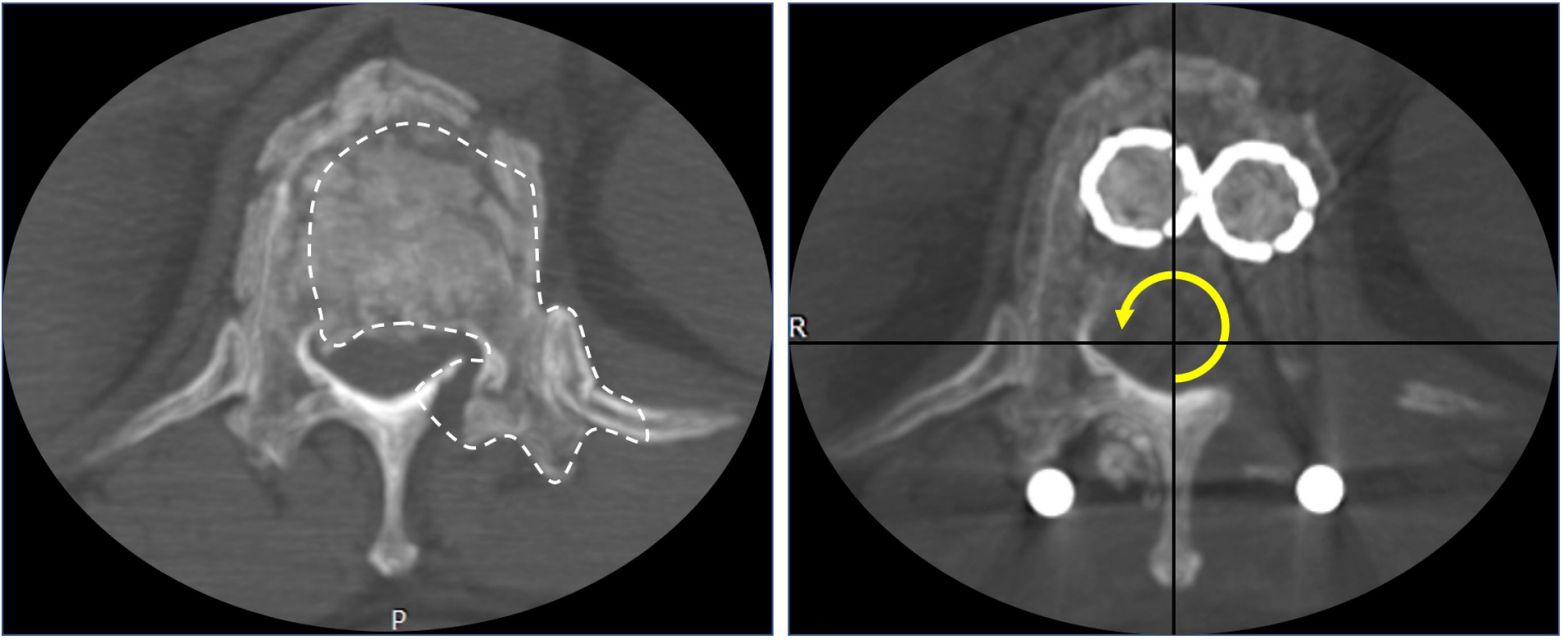
Transverse images of pre-operation and after 270° canal decompression. The white dotted line represents the range of decompression and the yellow arrow represents the decompressed area in 270°.

Intraoperative data, including operative time, estimated blood loss, and surgery-related complications, were recorded. Thin-cut computed tomography and x-ray were performed postoperatively in all patients (Figure 5). The patients were asked to wear a thoracolumbar brace for three months after surgery. Routine follow-ups were performed at 6 weeks and 3, 6, 12, and 24 months postoperatively. When bone fusion was observed on computed tomography examination, the patient was advised to undergo internal implant removal to retain non-fusion segment movement.

### Statistics

Continuous variables were expressed as mean ± standard deviation and range. The comparison of these variables were conducted using repeated measurements with a Wilcoxon signed-rank test. Categorical variables of interest were presented as percentages. All statistical analyses were performed in SPSS, version 25.0 (SPSS, Inc.). The statistical significance threshold was denoted as P values <0.05.

## Results

Preoperative measurements showed that the average preoperative canal compromise and anterior body height compression were 58.4% and 50.5%, respectively. All surgeries were successfully completed in one phase. Twenty-four patients underwent corpectomy with contralateral laminae, and spinous processes remained. Three patients underwent removal of the spinous process as required to reveal the dura sac for sufficient repair of the dural sac rupture due to severe burst fracture. However, one of the patients experienced cerebrospinal fluid leakage after dura sac repair. In all patients, neurological function recovery was significantly improved except for three grade A patients. The operation lasted 151.5 ± 25.5 min (range: 115–220 min), the estimated blood loss was 590.7 ± 169.9 ml (range: 400–1,000 ml), and the follow-up time was 25.8 ± 8.6 months (range: 18–48 months). Neurological function recovery was significantly improved in all patients except 3 grade A patients. No wound infection and severe neurovascular injury occurred. Two patients experienced pleural tears intraoperatively, but no pneumothorax occurred after closing the surgical incision and chest x-ray examination. Only one patient had an aggravation of neurological damage due to accidental injury when using the off-centered curette. The VAS for back pain was significantly decreased from 6.8 to 0.7 compared with the values pre-operation and at the last follow-up (*P* = 0.000). The correct deformity angle was 12.4° ± 4.7, and the anterior body height recovery was 96.7% postoperatively. The correct loss angle was 2.1° ± 1.0, and TMC subsidence was 1.3 ± 0.7 mm at the last follow-up. Defined TMC subsidence occurred in 3 patients (3/27) at the last follow-up. During follow-up, CT showed efficient 270° spinal canal decompression (Figure 5), no aggravation of thoracic and lumbar pain, no screw loosening or breakage, and no displacement of TMCs. Bony fusion was achieved in all patients. All the data are displayed in Table 2.

**Table 2:** Summary of surgical and clinical data.

Patients	Injury level	Operative time (mins)	Estimated blood loss (ml)	VAS		Neurological status (ASIA)		Canal Compromise	Anterior Body height compression	Anterior Body height recovery	Correct deformity angle (°)	Loss correct angle (°)	TMC Subsidence (mm)	Bone fusion
				Preop	FU	Preop	FU							
1	L1	210	800	7	0	C	E	45.9%	45.2%	96.9%	13.7	1.4	1.2	yes
2	L4	220	1,000	8	1	B	D	83.1%	48.5%	92.1%	3.9	2.9	1.7	yes
3	T12	150	400	6	0	B	D	47.0%	48.7%	100.4%	18.9	2.1	0.8	yes
4	T12	160	600	6	2	C	D	44.6%	35.4%	101.8%	15.6	3.6	1.5	yes
5	T12	170	600	8	1	C	E	52.2%	52.3%	96.7%	13	1.1	0.6	yes
6	T12	180	1,000	8	1	C	D	34.0%	41.0%	89.6%	10.4	1.2	1.3	yes
7	L1	170	600	6	2	D	E	35.9%	38.5%	101.3%	7	2.5	1.4	yes
8	L4	190	800	8	1	A	A	78.9%	36.0%	95.5%	13.2	2.2	1.5	yes
9	T12	130	450	6	1	C	D	38.8%	41.8%	99.8%	10.7	2.8	1.9	yes
10	L3	140	600	7	1	A	A	65.6%	47.7%	92.9%	16.1	2.7	1.3	yes
11	L2	135	400	7	0	C	E	55.4%	41.6%	101.0%	15.5	2.5	0.8	yes
12	T12	150	800	6	0	D	E	48.2%	38.8%	97.2%	15.9	2.3	1.7	yes
13	T12	155	600	8	0	B	D	64.1%	61.7%	93.5%	23.3	0.3	0.4	yes
14	L1	145	600	7	1	B	C	63.9%	54.6%	102.5%	16.4	1.3	0.5	yes
15	L1	160	600	7	0	A	A	77.6%	63.8%	91.3%	8.6	1.7	1.2	yes
16	T11	130	600	6	1	C	D	60.4%	67.2%	87.4%	16	4.2*	3.1*	yes
17	L1	125	400	6	0	C	D	51.7%	47.8%	101.6%	12	1.3	1.2	yes
18	T12	145	500	7	0	D	E	42.6%	38.4%	98.8%	8.8	1.3	0.8	yes
19	L2	125	400	6	0	C	D	61.8%	41.7%	97.2%	9.1	1.4	1.3	yes
20	T12	155	600	6	0	C	D	64.1%	67.5%	101.7%	18.6	2.4	1.7	yes
21	L1	140	600	7	2	A	C	80.2%	59.1%	104.6%	10.4	3.3	2.7*	yes
22	T12	150	600	7	1	C	D	59.3%	68.3%	92.9%	15.9	3.7	2.5*	yes
23	T11	130	400	7	1	D	E	57.3%	60.9%	92.8%	9.6	1.6	1.4	yes
24	T10	125	400	7	1	C	D	66.8%	58.3%	90.8%	14.2	1.4	0.7	yes
25	T12	140	600	6	0	C	D	57.3%	47.2%	99.6%	5.6	2.5	1.2	yes
26	L1	145	600	7	0	B	D	71.2%	48.7%	101.5%	8.5	0.1	0.3	yes
27	L3	115	400	7	1	C	D	69.9%	62.1%	90.7%	4.4	1.8	1.2	yes
Mean	/	151.5	590.7	6.8	0.7	/	/	58.4%	50.5%	96.7%	12.4	2.0	1.2	/

Preop, preoperation; FU, follow-up; VAS, visual analogue scale; ASIA, american spinal injury association; CSF, cerebrospinal fluid; TMC, titanium mesh cage.

* Represent significant subsidence.

## Discussion

“Burst fractures” were first described by Holdsworth (13) in the 1960 s as fractures caused by the axial load. They frequently occur due to high-energy trauma and are most associated with falling and traffic accidents (2). These fractures can cause neurologic complications and kyphotic deformities, and they represent 10%–20% of all spine fractures at or near the thoracolumbar junction (3).

The single-stage posterior transpedicular or costotransversectomy approach for corpectomy and reconstruction has gradually become popular for thoracolumbar burst fractures (3, 5, 6, 14, 15), because spinal surgeons are familiar with the posterior approach, and studies show that the posterior approach is associated with a relatively low risk of complications compared with anterior or combined anterior and posterior approaches. However, the placement of a large fusion cage may require the sacrifice of a nerve root in the thoracic spine due to its fixed height. Despite using expandable cages, some surgeons still amputate nerve roots to accommodate a larger cage for better anterior reconstruction (16). Cages with smaller diameters are often used in the lumbar spine to preserve nerve roots and prevent nerve injury, but they carry the risk of cage subsidence when the footplate-to-vertebral body endplate ratio is less than 0.5 (17). One research (18) showed that cage subsidence was observed in the early postoperative period in as high as 35% of patients, and subsidence was demonstrated to be higher in patients with expandable cages than in patients with non-expandable cages (17). A TMC is a non-expandable cage that is a good option for supporting the anterior spinal column after a corpectomy. The anterior column supports approximately 80% of the axial load, and its efficiency for reconstruction is crucial (19). Biomechanical studies have indicated that TMC is resistant to axial compression (20). These cages are available in different diameters and heights, and the tamping of autografts inside the mesh can significantly promote osteoinduction. Furthermore, TMCs can be easily trimmed for adaptation to sagittal inclination, and interdigitation of the cage with the vertebral body endplate provides primary stability.

The posterior approach often requires the full removal of posterior spinal attachment. However, additional posterior fusion can enhance late-stage stability and reduce the occurrence of non-fusion and cage subsidence at injured segments (17). Therefore, when spinal canal decompression is required, it is necessary to preserve the posterior structure of the spinal canal for bone graft fusion and to enhance the spinal stability after reconstruction. Finally, the vertebra was subjected to subtotal corpectomy with a unilateral approach to achieve 270° of canal decompression and three-column reconstruction using double TMC.

In our study, all patients suffered from thoracic and lumbar burst fractures with neurological damage. The preoperative spinal canal compromise and anterior body height compression were 58.4% and 50.5%. The indication for surgical decompression and reconstruction was necessary. We compared the results of previous studies on thoracic and lumbar burst fractures with our results (Table 3). The data showed that our technique had obvious advantages in terms of the operative time and estimated blood loss than the anterior approach. The incidence of complications was within an acceptable range, and the anterior body height was almost corrected. In terms of cage subsidence, the double cage shared the stress load from the upper and lower endplates. Theoretically, the bone contact area on one side of double TMC with a diameter of 16 mm was 402 mm^2^, and that of the single cage with a diameter of 22 mm was only 380 mm^2^. As a result, the double 16 mm cage unit area received less stress, and the larger bone contact area was better conducive to bone fusion under a theoretical condition.

**Table 3:** Comparison of different studies in treatment for thoracolumbar burst fractures.

Study Characteristic	Our study	Zahra et al., 2011	Lee et al., 2014		Kang et al., 2013	Sasani et al., 2009	Hofstetter et al., 2011	Yang et al., 2010	Suzuki et al., 2012
No. of patients	27	22	16	17	18	14	17	37	7
Mean age (years)	49.5	37.6	39.9	48.1	45	40.3	38.6	40.7	65
Sex (males/females)	22/5	17/5	9/7	13/4	/	6/8	7/10	34/3	5/2
Surgical approach	P	A	A	A	A	P	P	P	P
Cage	TMCs	TMC	TMC	EC	TMC	EC	EC	TMC	TMC
Operative time (min)	151.5	185	332	250	310	187	289	157	277
Estimated Blood loss (ml)	591	1,445	1,408	823	2,330	596	1,041	1,086	471
Mean follow-up time (months)	25.8	47.4	32.4	17.6	/	24	29.8	24	/
Complications rate (%)	14.8	/	/	/	/	/	23.5	12.5	/
No. of corpectomy	1	1	1	1	/	1	1	1	1
No. of levels instrumented	≥4	2	/	/	/	≥4	2–6	2–4	2–4
Body height correction%	96.75	/	/	/	/	/	/	94.08	/
Correct deformity angle (°)	12.4	5.4	8.81	12.09	23.6	9.1	4.9	21.26	26.7
Loss Correct angle (°)	2	1.9	5.99	1.82	2.3		/	0.8	6.3
Subsidence (mm)	1.2	/	7.99	9.85	/	/	/	/	/
Neurotomy rate (%)	0	/	/	/	/	/	35.3	/	/
Fusion rate (%)	100	/	/	/	100	/	/	100	/

P, posterior; A, anterior; TMC, titanium mesh cage; EC, expandable cage; “/” indicates missing data.

Intervertebral fusion has been proven to be effective in promoting the final biomechanical stabilization of the fracture and protecting the fixation instruments from material fatigue failure (21). Rapid and firm bone fusion requires good surface contact and stability in the fusion region. It has been proven that the using of anterior fixation alone or short posterior fixation alone after corpectomy and TMC implantation provides inadequate stability, which may be associated with the loss of correction and subsidence (12). Furthermore, significant cage subsidence and angular change lead to the loss of this stability and allow progressively more flexion-extension motion (22). Therefore, posterior fixation was applied with four points of fixation superior and inferior to the level of decompression in our study. Our results showed a satisfactory fusion rate of 100% and no screw loosening or breakage at the last follow-up. However, three cases had significant cage subsidence over 2 mm, and one had a combined loss correct angle over 4°. It seemed to be more likely to occur in patients with osteoporosis. Fortunately, none of the patients required two-stage or revision surgery.

**Figure 6:**
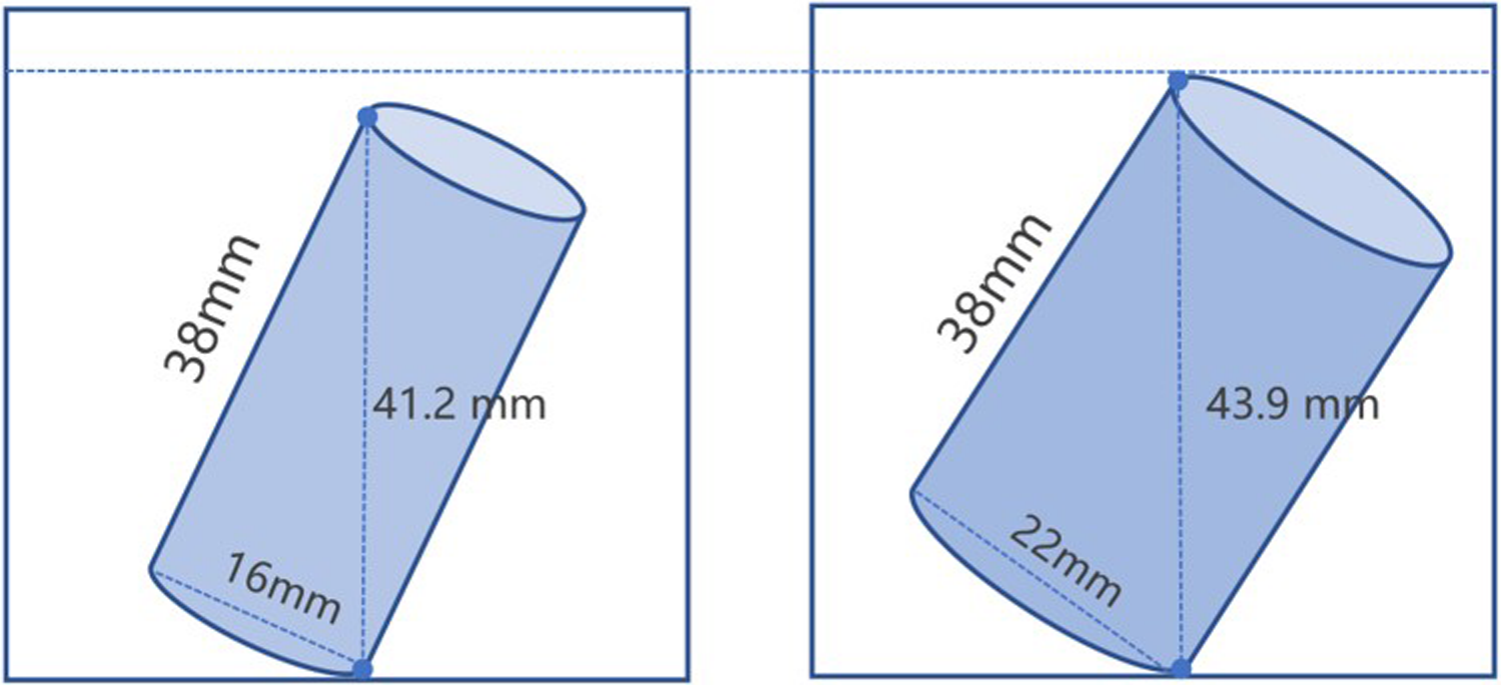
The schematic picture of tilted height. (**A**) shows the tilted height of 16 mm diameter TMC. (**B**) shows the tilted height of 22 mm diameter TMC.

In terms of surgical techniques, TMC insertion is technically demanding, but double cage with 16 mm diameters had no difficulty in terms of insertion. As we know, the larger the diameter of the cage was, the higher the tilted height was. The tilted height was the maximal height when a cylinder rotated from a horizontal to an upright position (Figure 6). Therefore, a single cage with a large diameter should be shorten the height, or the intervertebral space should be more axially distracted. Otherwise, the endplate may be damaged when tamping the cage. As a comparison, the small cage was more convenient to insert and effectively avoided surrounding tissue damage during insertion. Additionally, the double TMC with off-center positions were relatively closer to the cortical rim of the vertebral body, which was the strongest part of the endplate, thus reducing the chances of cage subsidence (23).

**Figure 7:**
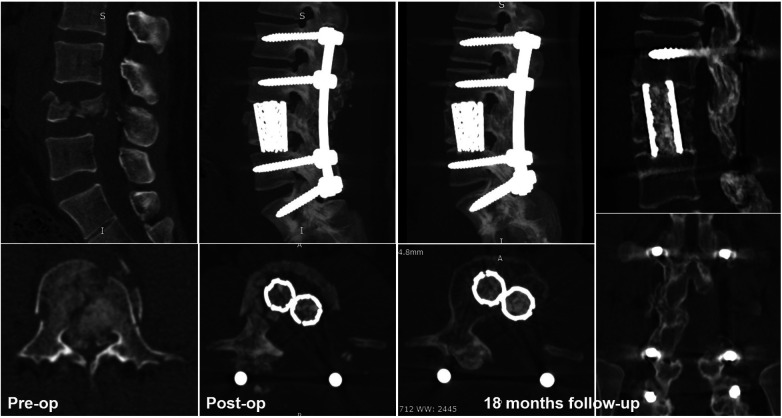
Typical case of intraoperative dural rupture, removal of unilateral laminae, and spinous processes for repair and completed 270° canal decompression and three-column reconstruction.

The posterior approach allowed for simultaneous treatment of both anterior and posterior structural elements. In the case of dural sac rupture, the posterior approach might be more convenient for dural sac repair, and 3 cases were successfully repaired during the operation in our study (Figure 7). To accommodate the passage of a large supporting construct when cage insertion, the surrounding nerve elements have a risk of injury. Our technique made up for the shortcomings of the posterior approach. Double TMC were easier to place and obviously shortened the operative time with nerve root preservation. However, our study still had several shortcomings that we need to emphasize. First, decompression of the contralateral side is technical demand and the performance of this technique requires an experienced surgeon. Second, this study is retrospective, and previous cases with one TMC will be enrolled for a Case-control study. Third, the number of included samples in this study is limited, so further technical improvement and experience summary needs to be carried out continuously in a larger sample. Finally, the technique is modified from the traditional posterior approach, and it supplies an alternative method for surgeons when treated with similar fractures.

## Conclusion

The posterior unilateral approach with 270° spinal canal decompression and three-column reconstruction using double titanium mesh cages is a clinically feasible, safe and alternative treatment for thoracic and lumbar burst fractures.

## Data Availability

The original contributions presented in the study are included in the article/Supplementary Material, further inquiries can be directed to the corresponding author/s.
